# Climate Change Enhances the Cultivation Potential of *Ficus tikoua* Bur. in China: Insights from Ensemble Modeling and Niche Analysis

**DOI:** 10.3390/biology14111473

**Published:** 2025-10-23

**Authors:** Mei Liu, Yutong Qin, Jian Yang, Xiaoyu Li, Fengli Zhu, Zhiliang Ma, Cong Zhao, Ruijun Su, Yan Chen

**Affiliations:** 1 School of Chemistry and Materials Engineering, Mianyang Normal University, Mianyang 621000, China; liumei@mtc.edu.cn; 2 School of Life Sciences (School of Ecological Forestry), Mianyang Normal University, Mianyang 621000, China; qinyutong@stu.mtc.edu.cn (Y.Q.); lixiaoyu@stu.mtc.edu.cn (X.L.); zhufengli@stu.mtc.edu.cn (F.Z.); 3 Sichuan Provincial Forest and Grassland Key Laboratory of Alpine Grassland Conservation and Utilization of Tibetan Plateau, College of Grassland Resources, Southwest Minzu University, Chengdu 610041, China; yjian@swun.edu.cn; 4 Key Laboratory of Southwest China Wildlife Resources Conservation (Ministry of Education), College of Life Sciences, China West Normal University, Nanchong 637009, China; feng281@126.com; 5 School of Environmental Science and Engineering, Southwest Jiaotong University, Chengdu 611756, China; zhaocongmy@my.swjtu.edu.cn

**Keywords:** *Ficus tikoua* Bur., climate change, ensemble modeling, species distribution model, niche conservatism

## Abstract

**Simple Summary:**

*Ficus tikoua* Bur. is a traditional plant in Southwest and South China, valued for its food, medicinal, and ecological functions, including soil and water conservation. We used computer models to predict where this plant can grow now and in the future under climate change. Currently, it is primarily suited for the Yunnan–Guizhou Plateau and the Sichuan Basin. In the coming decades, warming temperatures may allow the plant to spread to higher latitudes and elevations in the northwest. At the same time, some southeastern areas may become less suitable due to heat stress. Overall, our results suggest that climate change may increase opportunities to cultivate *F. tikoua* in new regions, while also emphasizing the importance of protecting existing core habitats and monitoring vulnerable edge areas.

**Abstract:**

Climate change is reshaping plant distribution and ecological adaptation worldwide. *Ficus tikoua* Bur., a perennial resource plant native to Southwest and South China, has not been systematically assessed for its future cultivation potential. In this study, we used the Biomod2 ensemble modeling framework, integrating 12 algorithms with 469 occurrence records and 16 environmental variables, to predict the potential distribution and niche dynamics of *F. tikoua* under current and future climate scenarios (SSP126, SSP370, and SSP585). The ensemble model achieved high predictive accuracy based on multiple algorithms and cross-validation. The minimum temperature of the coldest month (bio6, 43.5%), maximum temperature of the warmest month (bio5, 25.0%), and annual precipitation (bio12, 10.3%) were identified as the dominant factors shaping its distribution. Model projections suggest that suitable habitats will generally expand northwestward, while contracting in the southeast. Core areas, such as the Yunnan–Guizhou Plateau and the Sichuan Basin, are predicted to remain highly stable. In contrast, southeastern marginal regions are likely to experience a decline in suitability due to intensified heat stress. Niche analyses further revealed strong niche conservatism (overlap D = 0.83–0.94), suggesting that the species maintains stable climatic tolerance and adapts primarily through range shifts rather than evolutionary change. This finding suggests limited adaptive flexibility in response to rapid warming. Overall, climate warming may enhance cultivation opportunities for *F. tikoua* at higher latitudes and elevations, while emphasizing the importance of protecting stable core habitats, planning climate adaptation corridors, and integrating this species into climate-resilient agroforestry strategies. These findings provide practical guidance for biodiversity conservation and land-use planning, offering a scientific basis for regional policy formulation under future climate change.

## 1. Introduction

Global climate change is reshaping species distributions and ecosystem structures, posing major ecological and socioeconomic challenges in the Anthropocene [1,2]. Predicting potential distribution shifts under future climates has become a central focus in ecology and sustainable agricultural planning [3,4,5]. Species Distribution Models (SDMs) have been widely applied to biodiversity conservation, as well as the spatial planning of crop and forestry introduction and cultivation [6,7,8], as they integrate species occurrence data with multidimensional environmental variables to infer potential ecological niches and geographic distributions quantitatively. Recently, the ensemble modeling approach has mitigated the uncertainties of single-model predictions, offering a robust framework for analyzing complex ecological relationships with enhanced accuracy and robustness [9,10]. Similar techniques have been successfully applied to assess potential distribution shifts in subtropical and agricultural plants such as *Ficus carica* [11], *Camellia oleifera* [12], and *Oryza sativa* [13], providing methodological references for this study.

In this context, evaluating the responses of regionally significant species to climate change is essential for informing conservation strategies and promoting sustainable cultivation practices. *Ficus tikoua* Bur. (family Moraceae, genus *Ficus*) is a typical prostrate perennial plant that is widely distributed in subtropical and warm-temperate regions of Southwest to South China [14]. This species is integral to soil retention, water conservation, and ecosystem stability, while also holding substantial ecological and economic significance due to its traditional use as food and medicine in local communities [14,15]. Its rhizomatous growth further enhances its potential for sustainable cultivation and supports rural livelihoods. However, its natural distribution is constrained by climatic thresholds, including frost damage from low temperatures and stress from extreme summer heat, which creates distinct boundary effects.

Compared with other subtropical *Ficus* species, *F. tikoua* exhibits distinctive traits that enhance its significance as both a study subject and a resource. Its creeping, mat-forming growth habit provides superior soil stabilization, which is particularly valuable under increasing extreme rainfall events [16]. More importantly, its sensitivity to cold (bio6) and heat (bio5) extremes offers a measurable physiological indicator for assessing climate impacts, unlike congeners with broader thermal tolerances [16]. Additionally, its perennial rhizomes serve as nutrient reservoirs, conferring drought resilience and highlighting its potential as a sustainable candidate for starch production and climate-resilient agroforestry [16]. These unique attributes position *F. tikoua* as an ideal model species for biogeographic analysis and climate-adaptive cultivation research. Nevertheless, studies on its cultivation zoning and suitability mapping remain scarce, and systematic, macro-scale assessments of its potential distribution and habitat dynamics under future climate change scenarios are still lacking [17,18].

Numerous studies have demonstrated that temperature extremes and precipitation regimes are the primary determinants of subtropical plant distribution [19,20]. For instance, extreme cold constrains the northern and high-altitude boundaries of many woody species. In contrast, intense summer heat frequently drives the degradation or even local extinction of populations at their southern margins [21,22]. Similarly, the distribution of *Ficus tikoua* is highly sensitive to climatic extremes. Low winter temperatures frequently restrict its ability to overwinter and the survival of its rhizomes at higher latitudes, while excessive summer heat and drought can inhibit stolon elongation and reduce rhizome carbohydrate accumulation [23,24]. These climatic constraints collectively define its current range boundaries and render it responsive to variations in temperature and precipitation patterns. These ecological patterns suggest that the potential distribution of *F. tikoua* under future climate change may undergo spatial reorganization, characterized by northwestward expansion and southeastward contraction. However, there is currently a lack of quantitative predictions based on ensemble models, as well as a comprehensive assessment of their future cultivation potential and ecological fate combined with niche conservatism analysis.

Therefore, based on 469 valid distribution records of *F. tikoua* and 16 key environmental variables, this study employed an ensemble modeling framework and niche analysis to assess its potential geographic distribution and climatic suitability patterns, as well as centroid migration and niche stability in China under current and future (SSP126, SSP370, and SSP585) climate scenarios. The main research objectives were as follows: (1) to identify the core environmental factors governing the distribution pattern of *F. tikoua* and their ecological mechanisms; (2) to reveal the dynamic changes and migration directions of the potential suitable habitats of *F. tikoua* under climate change; and (3) to evaluate the future stability of *F. tikoua*’s niche and its implications for cultivation zoning and resource utilization. This study not only provides a scientific basis for promoting *F. tikoua* cultivation and adjusting regional agricultural structures in China, but also offers new evidence for understanding the distribution response mechanisms and ecological adaptation strategies of subtropical plants under Anthropocene climate change.

## 2. Materials and Methods

### 2.1. Species Distribution Records

**Figure 1:**
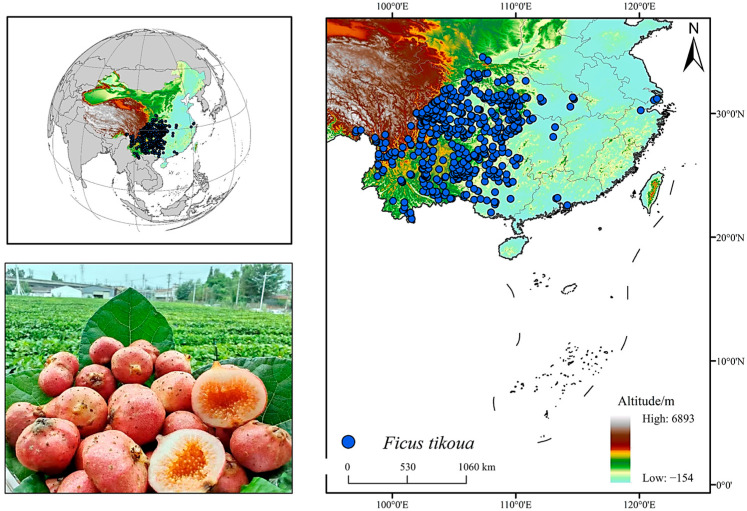
Geographic distribution of *F. tikoua*.

A total of 751 distribution records of *F. tikoua* were collected in this study by accessing the National Plant Specimen Resource Center (NPSRC, http://www.cvh.ac.cn/, accessed on 15 July 2025). All records were cross-checked to remove duplicates, erroneous coordinates, and misidentified specimens. To prevent model overfitting caused by spatial clustering, one record per 5 × 5 km grid cell was retained. This spatial resolution was selected to balance the scale of environmental layers with the clustering pattern of occurrence points, effectively reducing spatial redundancy while avoiding excessive data loss. Ultimately, 469 valid distribution records were obtained (Figure 1).

### 2.2. Selection and Processing of Environmental Variables

A total of 41 environmental variables were initially considered, including 19 bioclimatic factors, 16 soil properties, three topographic variables, and three indices representing human and vegetation influences (Human Footprint Index, land use type, and NDVI). Current and future climate data were downloaded from the WorldClim database (http://worldclim.org/data/index.html, accessed on 15 July 2025). Future climate projections were based on three Shared Socioeconomic Pathway (SSP) scenarios (SSP126, SSP370, and SSP585), representing low-, intermediate-, and high-emission pathways, respectively [25,26]. Soil and topographic data were derived from the Harmonized World Soil Database (HWSD) of the Food and Agriculture Organization (FAO) (http://www.fao.org/faostat/en/#data, accessed on 15 July 2025). All variables had a spatial resolution of 2.5 arc-minutes (covering approximately 25 km^2^).

To reduce redundancy arising from multicollinearity, a two-step variable selection procedure was employed before model construction. First, all environmental variables were input into the ensemble model for three initial runs, and variables with zero contribution rates were excluded. Second, the remaining variables were subjected to Spearman correlation analysis, and where the correlation coefficient between two variables was ≥0.8, the variable with the highest contribution rate was retained [26,27]. Following this screening process, 16 environmental variables were retained, including six climatic factors, five soil factors, three topographic factors, and two anthropogenic/vegetation-related factors (Table 1). Although some variables contributed less than 3% to the final model, they were retained to maintain ecological completeness and capture potential synergistic effects with dominant climatic variables. Such minor predictors can improve the realism of ensemble outputs without substantially increasing model complexity.

These retained variables collectively represent the key ecological constraints shaping the distribution of *Ficus tikoua*. Climatic variables (e.g., bio5, bio6, bio12) define the species’ temperature and moisture thresholds, which are closely linked to its sensitivity to frost and drought stress. Topographic factors (altitude, slope, and aspect) influence local microclimate and soil moisture availability, thereby affecting stolon growth and rhizome survival on sloping terrains. Soil parameters (e.g., pH, organic carbon, and available water capacity) determine nutrient conditions and substrate suitability for root development. In addition, NDVI and land-use/land-cover indices capture habitat productivity and human disturbance intensity, both of which influence the establishment and persistence of *F. tikoua* populations.

To ensure the robustness of variable selection, the preliminary ensemble runs were conducted using three representative algorithms, Random Forest (RF), Generalized Additive Model (GAM), and MaxEnt, which capture nonlinear, additive, and presence-only modeling structures, respectively. Running three iterations allowed us to stabilize contribution rankings while avoiding computational redundancy, as further repetitions produced negligible changes (<1% variation) in variable importance. This two-step procedure has been adopted in similar ensemble modeling studies [28,29], ensuring both efficiency and transparency in variable screening.

### 2.3. Construction and Expression of Ensemble Models

The Biomod2 platform integrates 12 species distribution algorithms and allows for the construction of ensemble models by combining species occurrence data with pseudo-absence data. In this study, pseudo-absence points were generated from background data using the random selection method, with 1000 pseudo-absence points created for each run to balance model stability and computational efficiency. Model parameters were optimized using the “biomod-tuning” function, with 75% of the records randomly selected for model training and the remaining 25% reserved for independent validation. To avoid bias, the weights of the presence and pseudo-absence data were set equal. This procedure was repeated 10 times, resulting in 100 simulations across 10 replicate runs.

Model performance was assessed using three commonly adopted evaluation metrics: the Area Under the Receiver Operating Characteristic Curve (AUC), Cohen’s Kappa (KAPPA), and the True Skill Statistic (TSS). Following the established criteria [30,31], only models with TSS ≥ 0.7, which indicates “good to excellent” predictive accuracy, were retained for ensemble construction. The final ensemble model was generated using a weighted mean approach (EMwmean), where individual model weights were proportional to their TSS scores, thereby ensuring a greater influence from models with higher performance.

To translate continuous habitat suitability values into ecologically interpretable classes, a 0/1 cutoff threshold was applied to distinguish suitable from unsuitable areas. Prediction probabilities were then reclassified into four suitability categories: high suitability (0.8 ≤ P ≤ 1.0), moderate suitability (0.5 ≤ P < 0.8), low suitability (0.25 ≤ P < 0.5), and unsuitable (P < 0.25). Finally, the spatial patterns of habitat suitability were mapped and visualized using ArcGIS v10.4.1.

### 2.4. Niche Changes

To evaluate whether *F. tikoua* exhibited niche shifts under future climate scenarios, we performed ecological niche analyses using the ecospat package (v4.1.1) in R v4.3.2. Habitat suitability outputs from the current and projected scenarios were converted into binary presence–absence maps (0/1) based on ensemble model thresholds. We then calculated Schoener’s D index to quantify niche overlap between time periods, where values close to 1 indicated high overlap, and values near 0 suggested little to no overlap. To statistically assess niche stability, niche equivalence, and niche similarity, tests were conducted through repeated randomization.

### 2.5. Centroid Migration Patterns

The geographic coordinates of habitat centroids were computed using the R package SDMTools (v1.1-21), based on binary suitability maps. The latitude and longitude of each centroid, as well as the Euclidean distance of centroid displacement between scenarios, were then extracted and quantified using ArcGIS v10.4.1. In addition, raster-based suitability change maps were visualized in ArcGIS to illustrate both the direction and magnitude of centroid migration.

## 3. Results

### 3.1. Model Accuracy

This study systematically evaluated the accuracy and stability of common species distribution models in predicting the potential distribution of *F. tikoua* using three metrics: Kappa, AUC, and TSS. The data analysis revealed significant differences in performance among the models. The RF model exhibited excellent predictive ability, with a Kappa value of 0.9968, an AUC of 1.0000, and a TSS of 0.9965; the extremely low standard deviation indicated high stability. Two ensemble learning algorithms, Extreme Gradient Boosting (XGBoost) and Gradient Boosting Machine (GBM), also performed well. XGBoost achieved Kappa, AUC, and TSS values of 0.8840, 0.9783, and 0.9195, respectively, which were significantly superior to those of traditional statistical models.

**Figure 2:**
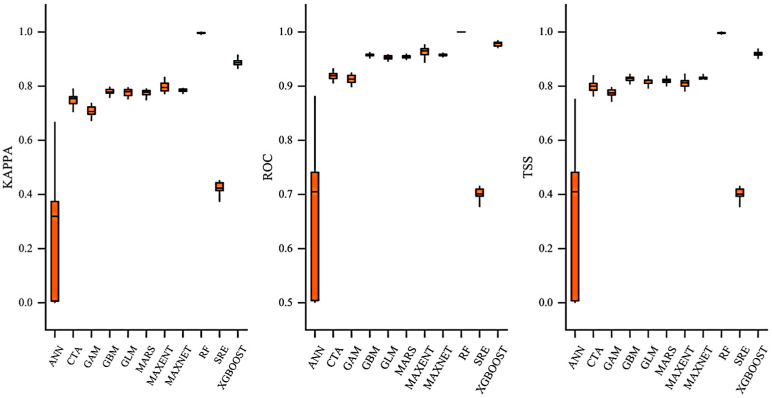
Model performance evaluation of 11 algorithms implemented in Biomod2. Boxplots show the distribution of Kappa, the Area Under the Receiver Operating Characteristic Curve (AUC), and true skill statistics (TSS) values across replicate runs for each model.

Notably, the analysis of the standard deviation of each model revealed important stability characteristics (Figure 2). The RF model had a standard deviation of less than 0.003 for all metrics, indicating highly consistent performance across different data subsets. In contrast, the Artificial Neural Network (ANN) had a Kappa standard deviation as high as 0.2253 and a TSS standard deviation of 0.1126, suggesting significant instability in its parameter optimization process, possibly due to the vanishing gradient problem under small-sample training. Among traditional statistical models, the Generalized Additive Model (GAM) had a Kappa standard deviation of 0.0862, showing moderate stability.

Based on these results, the ensemble model constructed in this study outperformed all single models, with TSS (0.960 ± 0.019), Kappa (0.931 ± 0.011), and AUC (0.997 ± 0.002) achieving near-perfect predictive accuracy and extremely low standard deviations (≤0.001 for all metrics). This confirms that the ensemble method effectively avoids the structural biases of single models by integrating the advantages of multiple algorithms, achieving an optimal balance in the bias-variance tradeoff.

### 3.2. Environmental Factors Influencing the Geographic Distribution of F. tikoua

Analysis of the contribution rate of environmental variables in species distribution models serves as a key basis for identifying the dominant ecological factors [32]. Currently, there is no unified standard in the academic community for determining the number of dominant factors; most studies adopt the cumulative contribution rate threshold method, that is, selecting the top several variables whose cumulative contribution rate reaches a specific value (usually 70–90%). Still, the specific threshold needs to be subjectively defined based on the ecological characteristics of the species [33]. Drawing on the niche conservatism hypothesis and variable significance tests, this study defines environmental factors with an individual contribution rate of 10% or greater as dominant environmental factors [8]. Analysis of the contribution rate of environmental variables based on the output of the ensemble model (Table S1) shows that among all 16 environmental variables, the minimum temperature of the coldest month (bio6, 43.5%), maximum temperature of the warmest month (bio5, 25.0%), and annual precipitation (bio12, 10.3%) all have contribution rates exceeding 10%, with a total cumulative contribution rate of 78.8%. These three variables were the dominant factors determining the potential distribution pattern of *F. tikoua*. Followed by the annual temperature range (bio7, 6.4%) and soil available water capacity (awc_class, 5.3%), the contribution rates of the remaining environmental variables were all less than 3%. Therefore, this study identified bio6, bio5, and bio12 as the dominant factors influencing the potential distribution of *F. tikoua*.

**Figure 3:**
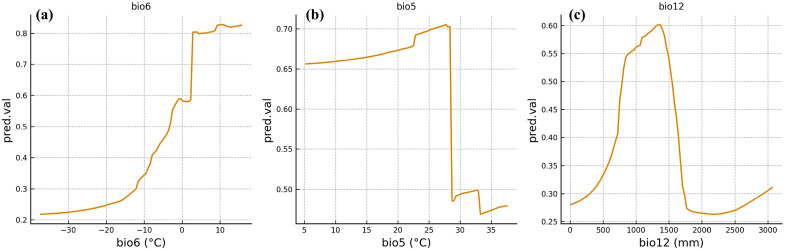
Environmental variable response curve.

The response curves output by the ensemble model reflected the suitable ranges of *F. tikoua* for key climatic variables (Figure 3a–c). For the minimum temperature of the coldest month (bio6), the species’ survival probability reached the highly suitable habitat threshold at a temperature of −1.5 °C and increased rapidly with rising temperatures, peaking at approximately 0.8 °C at 3.2 °C; thereafter, the growth trend slowed down. Thus, the suitable range of bio6 is approximately −1.5 °C to 6.2 °C. Along the gradient of the maximum temperature of the warmest month (bio5), the survival probability exceeds the highly suitable threshold when the temperature reaches 28.4 °C and peaks at approximately 0.7 °C at 32.1 °C; when the temperature exceeds 35.6 °C, the probability decreases significantly. The suitable range of bio5 is 28.4–35.6 °C. The response curve of annual precipitation (bio12) exhibits a unimodal distribution: the survival probability exceeds the highly suitable threshold at 850 mm, peaks at approximately 0.65 at 1250 mm, and then decreases gradually. When precipitation exceeds 1650 mm, the survival probability falls below the highly suitable threshold. The suitable range for bio12 is 850–1650 mm. In summary, the potential distribution of *F. tikoua* is primarily controlled by extreme temperature values (bio5 and bio6) in combination with total precipitation (bio12), with temperature factors playing a more prominent role, as they determine both the core distribution range and boundaries of the species’ suitable habitats.

### 3.3. Potential Geographic Distribution of F. tikoua Under Current Climatic Conditions

**Figure 4:**
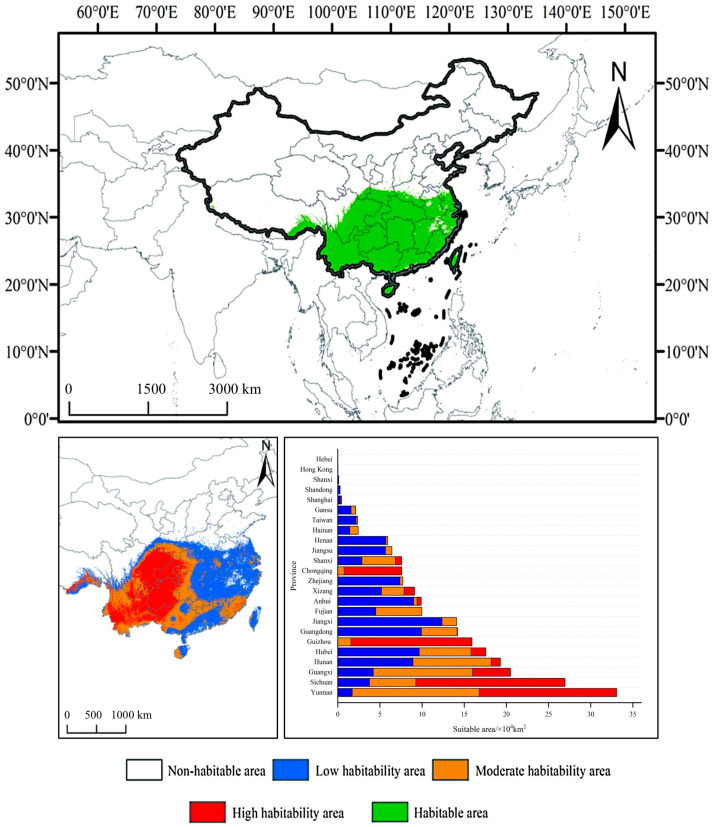
Current suitable habitat distribution of *F. tikoua*.

Based on the prediction results of the ensemble model (Figure 4), the total area of suitable habitats for *F. tikoua* in China is 86.74 × 10^4^ km^2^, primarily concentrated in the transition zone from subtropical to warm temperate regions (23–32° N, 98–122° E). The area of highly suitable habitats (P ≥ 0.8) is 28.15 × 10^4^ km^2^, distributed in continuous concentrated patches across the Yunnan–Guizhou Plateau (16.30 × 10^4^ km^2^ in Yunnan, 14.35 × 10^4^ km^2^ in Guizhou) and the western Sichuan Basin (17.71 × 10^4^ km^2^), which is highly consistent with the known actual distribution range of the species. The area of moderately suitable habitats (0.5 ≤ P < 0.8) is 32.67 × 10^4^ km^2^, distributed in a zonal pattern surrounding the highly suitable areas in Guangxi (11.77 × 10^4^ km^2^), Hunan (9.28 × 10^4^ km^2^), and Hubei (6.15 × 10^4^ km^2^). Lowly suitable habitats (0.25 ≤ P < 0.5) are widely but sparsely distributed across most provinces south of the Yangtze River, with larger areas in Jiangxi (12.37 × 10^4^ km^2^), Guangdong (9.92 × 10^4^ km^2^), and Hunan (8.92 × 10^4^ km^2^).

Notably, although certain areas in southeastern coastal provinces (Fujian, Zhejiang, Taiwan) and parts of central China (Anhui, Jiangxi) are suitable, the lack of highly suitable patches is constrained by the high-temperature and high-humidity environment (bio5 > 35.6 °C, bio12 > 1650 mm). Overall, the predicted spatial pattern of “highly–moderately suitable” habitats (with the Yunnan–Guizhou Plateau as the core and the Sichuan Basin as a secondary center) is highly consistent with the biological characteristics of *F. tikoua* (preferring warm and humid conditions but intolerant of waterlogging) and historical specimen records, confirming the reliability of the model in simulating distributions at the macro scale. In addition to climatic factors, soil and topographic variables also exerted secondary yet ecologically meaningful effects on the potential distribution of *F. tikoua*. Higher soil organic carbon and moderate pH values favored root and rhizome development, supporting its occurrence on well-drained loamy slopes. Elevation and slope influenced local temperature and moisture regimes, restricting the species to warm foothill zones and gentle inclines. Although the ensemble predictions captured these broad environmental gradients, spatial uncertainties may still arise from the coarse resolution of environmental layers and algorithmic differences among models. Nevertheless, ensemble averaging mitigates such projection artifacts, providing a more stable estimation of habitat suitability.

### 3.4. Prediction of Climate Change Impacts on the Potential Geographic Distribution of F. tikoua

**Figure 5:**
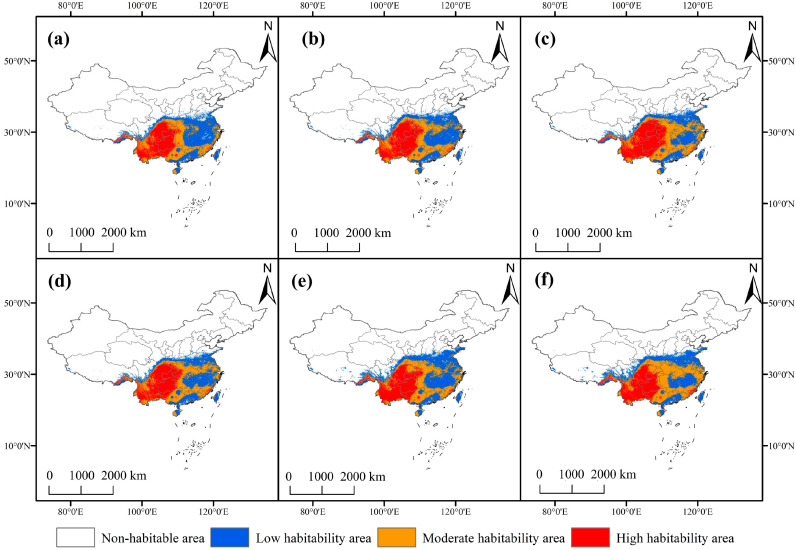
Changes in the potential geographic distribution of *F. tikoua* for the future climate change scenarios. Potential geographic distributions under different climate scenarios: SSP1-2.6 (**a**,**d**), SSP3-7.0 (**b**,**e**), and SSP5-8.5 (**c**,**f**). Potential geographic distributions in different periods: the 2050s (**a**–**c**) and the 2090s (**d**–**f**).

Based on the simulation results of the ensemble model for future climate scenarios (SSP1-2.6, SSP3-7.0, and SSP5-8.5) (Figure 5), the suitable habitat area of *F. tikoua* exhibited an overall expansion trend, with the expansion magnitude increasing as the radiative forcing intensified. Under current climatic conditions, the total area of suitable habitats is 233.69 × 10^4^ km^2^; by the 2090s under the SSP5-8.5 scenario, this area will increase to 282.03 × 10^4^ km^2^ (an increase of 20.7%). Specifically, the area of high-suitable habitats will grow from 65.09 × 10^4^ km^2^ (current) to 73.28 × 10^4^ km^2^ (2090s, SSP5-8.5), representing a 12.6% increase; the area of medium-suitable habitats will rise from 71.78 × 10^4^ km^2^ to 112.78 × 10^4^ km^2^ (a 57.1% increase); and the area of low-suitable habitats will decrease slightly from 96.82 × 10^4^ km^2^ to 95.97 × 10^4^ km^2^ (a 0.9% decrease).

Notably, despite the increase in the total suitable habitat area, the heterogeneity in responses to climate change among habitats of different suitability levels is significant. High-suitable habitats showed the slowest increase rate (12.6%) under the SSP5-8.5 scenario, while medium-suitable habitats exhibited the largest increase rate (57.1%) under the same scenario. This suggests that climate change may cause some high-suitable areas to be downgraded to medium-suitable areas, while originally non-suitable areas are converted to low- or medium-suitable areas. This phenomenon of “suitability level shift” is closely related to the niche edge effect driven by rising temperatures (increase in bio5) and changes in precipitation patterns (fluctuations in bio12).

From the perspective of spatial pattern evolution (Figure 4), the distribution area of *F. tikoua* shows a distinct bidirectional expansion characteristic (northwest-southeast). Under the SSP5-8.5 scenario, the newly added suitable habitat area in the 2090s will reach 48.48 × 10^4^ km^2^ (accounting for 20.8% of the current suitable habitat area), which will be mainly distributed in the Yangtze–Huaihe River Basin (28° N–32° N) north of the middle and lower reaches of the Yangtze River, and the western Yunnan–Guizhou Plateau (altitudes of 1500–2500 m). Meanwhile, the stability of the original suitable habitats remained high, ranging from 82.81% to 93.79% (Figure 4). The distribution centroid migrates approximately 120 km northwest, which is directly related to the relief of cold constraints caused by the increase in winter low temperatures (bio6). However, habitat loss has been observed in some southeastern coastal areas (e.g., the Zhejiang–Fujian Hills), with a maximum lost area of 0.58 × 10^4^ km^2^; this loss is likely due to extreme summer high temperatures (bio5 > 35.6 °C) exceeding the species’ physiological tolerance threshold. These shifts reflect the emergence of transitional zones where climatic conditions gradually approach the species’ tolerance thresholds, suggesting potential overlap between current and future suitable habitats. However, the actual range expansion may be constrained by dispersal capacity, habitat connectivity, and interspecific competition, meaning that realized migration could lag behind the projected climatic suitability. Overall, *F. tikoua* exhibits strong adaptation potential to climate change, but the reorganization of its distribution pattern suggests that differentiated conservation strategies may need to be developed in the future for emerging suitable areas in the northwest and vulnerable areas in the southeast. Although the ensemble model achieved high predictive accuracy, the present validation was limited to spatial cross-validation within the available dataset. Future work could incorporate independent temporal or regional datasets to assess the robustness of temperature-driven distribution patterns.

### 3.5. Analysis of Centroid Migration of Potential Suitable Habitats

**Figure 6:**
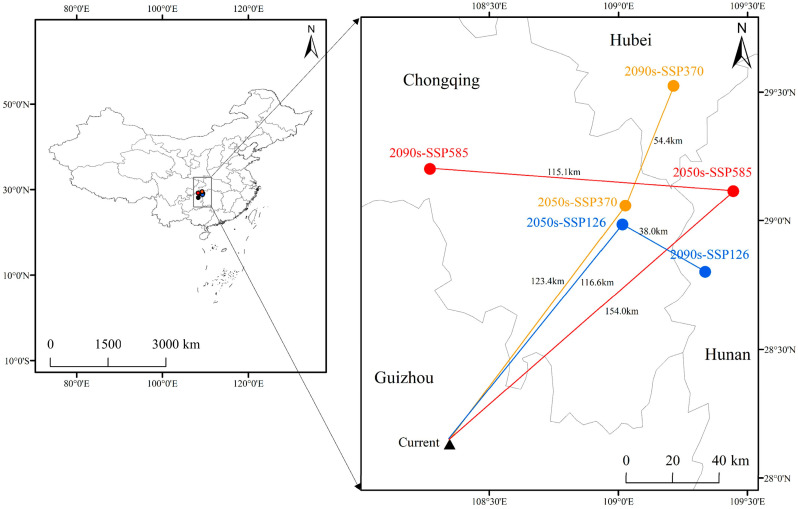
Changes in the gravity center of potential suitable habitat areas and the movement tendency of *F. tikoua* in future climate change scenarios.

Analysis of the spatial trajectory of the centroid of potentially suitable habitats (Figure 6) shows that under future climate scenarios, the distribution centroid of *F. tikoua* exhibits a significant northwestward migration trend, with the migration distance increasing as radiative forcing intensifies. The current distribution centroid is located at 28.73° N, 107.92° E (border area of Guizhou and Hunan Provinces). By the 2090s under the SSP5-8.5 scenario, the centroid will migrate to 29.18° N, 107.38° E (southern Chongqing), with a cumulative northwestward displacement of 54.4 km. In contrast, the migration distance under the SSP1-2.6 scenario during the same period was only 38.0 km, confirming the strong disturbance effect of high-emission scenarios on species distribution patterns.

This directional migration is highly consistent with the northwestward expansion of the temperature-limiting factor (bio6: minimum temperature of the coldest month) under climate warming. The low-temperature threshold that originally restricted the northern distribution of the species has been lifted by climate warming, leading to a shift in suitable habitats toward higher latitudes. Notably, the centroid migration trajectory shows spatial coupling with the topographic gradient (transition zone from the Yunnan–Guizhou Plateau to the Sichuan Basin), suggesting that the interaction between elevation and temperature jointly drives the species’ distribution response.

This finding suggests that although the total area of suitable habitats for *F. tikoua* may expand, the northwestward migration of its core distribution area may lead to the reorganization of the original ecosystem functions (e.g., disruption of symbiotic relationships with specific pollinators). Therefore, the construction of climate adaptation corridors should be proactively considered in conservation planning.

## 4. Discussion

### 4.1. Accuracy of the Ensemble Model and Reliability of Niche Modeling for F. tikoua

The predictive results of species distribution models are often jointly influenced by sample size, variable selection, and algorithmic differences, leading to high uncertainty [34,35]. For instance, small sample sizes tend to amplify outliers, thereby reducing the model’s generalization ability, while multicollinearity can lead to prediction bias and overfitting. These represent the limitations faced by many traditional studies when interpreting distribution patterns.

In this study, based on 469 valid distribution records, we constructed a weighted EMwmean by integrating multiple algorithms and introduced pseudo-absence point generation and multicollinearity testing to reduce uncertainty. The results showed that the ensemble model exhibited extremely high accuracy (AUC = 0.997 ± 0.002, TSS = 0.960 ± 0.019, Kappa = 0.931 ± 0.011), significantly outperforming the single models. By integrating the feature selection capability of RF, the residual optimization mechanism of XGBoost, and the smooth function fitting of GAM, the ensemble model effectively avoided the structural biases of single algorithms (e.g., the linear assumption limitation of GLM or the vanishing gradient problem of ANN), achieving an optimal balance between bias and variance, thereby obtaining the best predictive results. These findings not only verify the significant advantages of the ensemble model in species niche modeling but also provide a solid guarantee for the reliable simulation of *F. tikoua* distribution patterns in this study.

Combining the predictive results of the ensemble model constructed in this study with field distribution data, it was found that the high-suitability habitats of *F. tikoua* are mainly concentrated in parts of the Yunnan–Guizhou Plateau, Sichuan Basin, and Guangdong and Guangxi regions, which is highly consistent with its known natural distribution pattern (Figure 1). Specimen data and distribution records from the iPlant Database (https://www.iplant.cn) further verified the reliability of the predictive results. Previous studies have indicated that *F. tikoua* is primarily distributed in subtropical to warm-temperate regions in southwestern and southern China [36,37], which is consistent with the predictive results of this study. This indicates that the ensemble model not only performs excellently in terms of numerical accuracy but also closely matches real-world conditions in terms of spatial distribution patterns, thereby enhancing the credibility of the model’s predictive application for future climate change scenarios. In addition, although spatial thinning (5 × 5 km filtering) was applied to reduce sampling bias, residual spatial autocorrelation may still influence model calibration. Field-based validation of predicted suitable habitats was not available in this study, and future ecological surveys will be necessary to verify model predictions and assess local population persistence.

### 4.2. Ecological Significance of Dominant Environmental Factors and Their Shaping of Distribution Patterns

This study clearly revealed that bio6, bio5, and bio12 are the core factors determining the potential distribution pattern of *F. tikoua*, with their combined contribution rate reaching nearly 80%. Unlike previous studies that relied on mean annual temperature or annual precipitation to explain plant distribution, this study found that extreme temperature values are the rigid thresholds defining the distribution boundaries of *F. tikoua*, a result with significant ecological implications. Specifically, bio6 directly determined the overwintering survival capacity of *F. tikoua.* When the minimum temperature drops below −1.5 °C, plant tissues are prone to freeze damage [38], thereby restricting the species’ expansion to higher latitudes and alpine regions. In contrast, when winter temperatures remain within the range of −1.5 to 6.2 °C, the survival probability of *F. tikoua* increases sharply; this also constitutes the key climatic background for its stable distribution in the Yunnan–Guizhou Plateau and Sichuan Basin. Meanwhile, bio5 reflects the heat tolerance threshold and upper limit of photosynthetic efficiency of *F. tikoua*: when the temperature of the warmest month in summer exceeds 35.6 °C, photosynthesis is inhibited, and plants also suffer from severe transpiration stress [39,40], which limits the species’ distribution in the low-latitude arid-hot regions of South China. Therefore, extreme temperature values collectively formed the northern and southern distribution boundaries of this species.

Although the contribution rate of bio12 was slightly lower than that of the temperature factors, it played a key synergistic role in shaping the grades of suitable habitats. The model results show that *F. tikoua* exhibits the highest suitability within a precipitation range of 850–1650 mm, indicating that it possesses a certain degree of ecological plasticity and can tolerate a relatively wide range of moisture fluctuations. However, excessively low precipitation leads to drought stress, whereas excessively high precipitation (>1650 mm) may cause root anoxia and disease risks, thereby reducing habitat quality. This suggests that moisture factors alone are insufficient to determine the distribution boundaries; instead, they must interact with suitable temperature conditions to form stable high-suitability patches. Tiansawat et al. found that the distribution of *Ficus hispida* is also strongly constrained by extreme temperatures [41], with high summer temperatures significantly inhibiting photosynthetic efficiency. Meanwhile, controlled experiments on common subtropical broad-leaved trees conducted by Teskey et al. showed that moderate precipitation is a necessary condition for maintaining community stability [42]. Still, temperature thresholds are the decisive factors defining the outer edges of distribution. These results are highly consistent with the conclusions of this study, indicating that the distribution of *Ficus* species generally follows the rule of “dominance by extreme temperatures and synergistic regulation by moisture”. However, at the same time, the relatively wide suitable precipitation range of *F. tikoua* shows a certain uniqueness, allowing it to form continuous distributions across different moisture gradients in Southwest China. In summary, the potential geographic distribution of *F. tikoua* is typically driven by the combined effects of hydrothermal conditions. In contrast, the rigid factor of extreme temperature values mainly defines its distribution boundaries.

### 4.3. Climate Change-Driven Spatial Dynamics and Reorganization of Distribution Patterns

**Figure 7:**
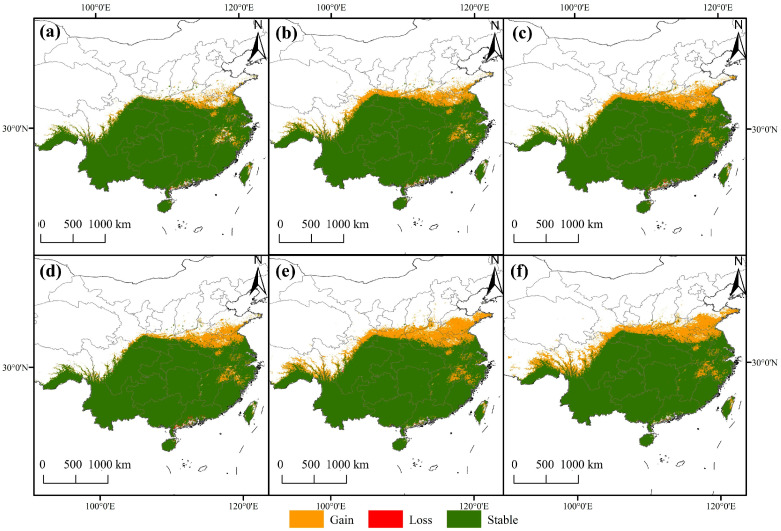
Changes in the potential geographic distribution of *F. tikoua* under climate change. Potential geographic distribution changes under different climate scenarios: SSP1-2.6 (**a**,**d**), SSP3-7.0 (**b**,**e**), and SSP5-8.5 (**c**,**f**). Changes in potential geographic distribution across different periods: the 2050s (**a**–**c**) and the 2090s (**d**–**f**).

The simulated results of the potential distribution of *F. tikoua* under future climate change revealed a typical spatial reorganization pattern characterized by expansion in the northwest and contraction in the southeast, a phenomenon consistent across different emission scenarios (Figure 7). The area of suitable habitats exhibited the largest expansion under the SSP585 scenario, increasing by approximately 20.7% by the 2090s. The newly added areas are primarily located on the northern margin of the Sichuan Basin, the Qinling-Huaihe River Basin, and the western margin of the Yunnan–Guizhou Plateau. This pattern is consistent with the migration trend of many subtropical plants toward higher latitudes and elevations under global warming [43,44], confirming the relaxation effect of “cold limitation” following the alleviation of low-temperature stress.

Notably, 38 km northwestward, the migration amplitude shows a positive correlation with the radiative forcing intensity. This directional migration is not only a direct response to climate warming but also reflects the coupling effect of climatic factors and topographic gradients. The transition zone from the Yunnan–Guizhou Plateau to the Sichuan Basin provides dual suitable conditions (temperature and elevation) for species expansion. This highlights that when predicting the future fate of a species, reliance on a single climatic variable is insufficient; instead, attention must be paid to the synergistic effects of topographic buffering and local climatic effects. However, decreased suitability or even localized loss of suitable habitats has occurred in parts of the southeastern coastal areas and South China. In contrast to the northwest expansion, southern marginal populations are constrained by extreme summer high temperatures (bio5 > 35.6 °C), exhibiting typical characteristics of climatic marginal effects. Previous studies have shown that extremely high-temperature events pose severe stress to the photosynthetic efficiency and water balance of tree species [42,45,46], which is highly consistent with the distribution of degraded suitable habitat areas in this study.

Therefore, the future distribution of *F. tikoua* is not a unidirectional expansion, but a complex process involving the coexistence of expansion, stability, and degradation. The stability of its core areas (the Yunnan–Guizhou Plateau and Sichuan Basin) exceeds 80%, while marginal areas show significant vulnerability. This spatial dynamic pattern has dual implications for ecosystem services and agricultural utilization. On the one hand, the newly emerging suitable areas in the northwest may serve as climate refugia and potential areas for future cultivation promotion. On the other hand, degraded areas in the southeast may face risks of community functional reorganization and declines in genetic diversity, necessitating strengthened monitoring and protection of marginal populations. Although the ensemble projections indicate an overall northwestward expansion, this reflects potential climatic suitability rather than actual colonization. The realized distribution of *F. tikoua* may be restricted by dispersal limitations, pollination specialization, and landscape fragmentation, which could hinder its migration into newly suitable habitats. However, the actual migration of *F. tikoua* may be further constrained by limited dispersal capacity and habitat fragmentation, potentially slowing or restricting colonization of emerging refugia. Moreover, the conservation implications discussed here are derived from bioclimatic projections rather than demographic or genetic evidence, and should therefore be interpreted as preliminary ecological inferences pending future field and population-level validation. Future studies integrating dispersal and land-use models would help refine these projections.

### 4.4. Implications of Niche Stability and the Future Fate of the Species

**Figure 8:**
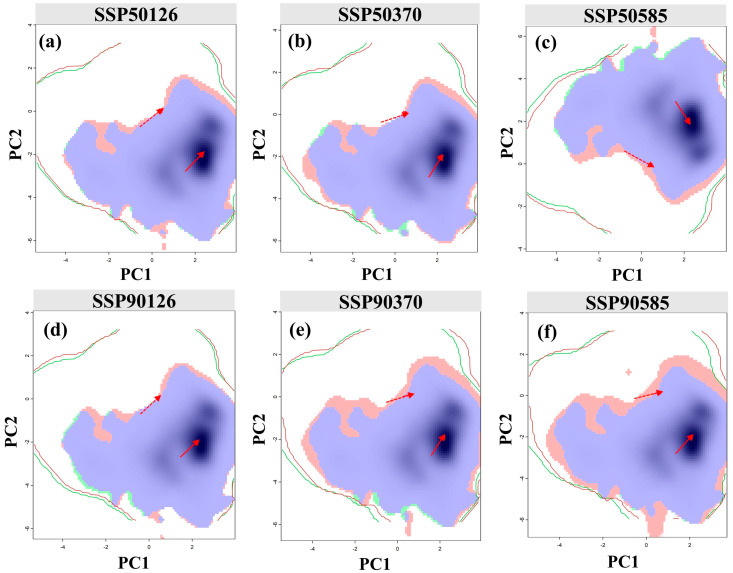
Dynamics and overlaps of the climatic niche for *F. tikoua* under climate change. The panels (**a**–**c**) show projections for the 2050s, and the panels (**d**–**f**) show projections for the 2090s, under the SSP1-2.6 (**a**,**d**), SSP3-7.0 (**b**,**e**), and SSP5-8.5 (**c**,**f**) scenarios. Green and red density contours represent species occurrence in current and future climates, respectively, with blue areas indicating niche overlap. Solid and dashed lines delineate 100% and 50% of the available environmental space, respectively. Red arrows track the direction and magnitude of the shift for the core climatic niche (solid line) and background center (dashed line).

The results of the niche analysis showed that *F. tikoua* exhibits significant niche conservatism under future climate change (Figure 8). Under the six scenarios, the niche overlap (Schoener’s D) remained at a high level of 0.83–0.94, and neither the niche equivalence test nor the niche similarity test rejected the null hypothesis. This suggests that a species’ environmental requirements and resource utilization patterns are essentially stable over time, and changes in their distribution are primarily driven by shifts in climatic space rather than niche evolution. This characteristic is consistent with the general conservatism of tropical and subtropical woody plants [47] and validates the methodological rationality of predictions based on SDMs.

This stability holds important ecological and conservation significance. First, *F. tikoua* can hardly rely on rapid evolution or niche shifts to adapt to climate warming; instead, it migrates to track its existing climatic preferences. However, this inference is based on bioclimatic modeling rather than empirical genetic or physiological evidence, and should therefore be regarded as a theoretical projection pending experimental verification. Future studies integrating population genetics, phenotypic plasticity, and physiological tolerance data would be valuable for clarifying whether *F. tikoua* primarily adapts or migrates under climate stress. This conclusion underscores the core role of “migration corridors” in future conservation. Specifically, climate adaptation corridors need to be established between the emerging suitable areas in the northwest and the core stable areas to ensure population connectivity and natural dispersal processes. From a management perspective, conservation efforts should further prioritize the protection of potential refugia in northwestern regions, restoration of habitat connectivity, and long-term monitoring of marginal populations to prevent genetic erosion and local extinction. Second, although the decline in suitability in the vulnerable areas of the southeast does not involve niche reorganization, it will cause a local population decline or even local extinction, which may have far-reaching impacts on genetic resource conservation and ecosystem functions.

Furthermore, niche conservatism suggests that the future fate of *F. tikoua* is more dependent on the rate and magnitude of climate change. If the rate of climate warming exceeds the species’ migration capacity, it may fall into the predicament of migration lag, leading to a mismatch between habitats and suitable environments. Similar phenomena have been observed in various tree species in previous studies [42], where the migration speed was insufficient to match the northward shift rate of climate zones. Therefore, the results of this study emphasize that in practical management, it is necessary to integrate in situ conservation and ex situ conservation: on the one hand, to maintain the stability of core areas, and on the other hand, to reserve potential expansion areas to ensure the long-term survival and utilization value of *F. tikoua* under future climate conditions.

## 5. Conclusions

Based on ensemble modeling and niche analysis, this study assessed how climate change may reshape the potential distribution and cultivation suitability of *Ficus tikoua* in China. The results indicate that temperature extremes and water availability jointly determine its range limits, leading to a general northwestward expansion and southeastern contraction under future scenarios. Core regions, such as the Yunnan–Guizhou Plateau and Sichuan Basin, are expected to remain relatively stable. The ensemble model performed robustly, though minor uncertainty is inevitable given the spatial resolution of input data and potential influences not explicitly represented in the modeling framework. Despite these limitations, the overall patterns provide valuable insights into the climate resilience of *F. tikoua* and its potential contribution to soil stabilization, biodiversity maintenance, and sustainable agroforestry systems in subtropical regions. From a management perspective, conservation strategies should prioritize the protection of stable refugia, restoration of habitat connectivity, and adaptive cultivation planning in emerging suitable areas to balance ecological conservation and rural development. Future research should integrate population genetics, physiological tolerance experiments, and long-term field validation to improve understanding of the species’ adaptive capacity and refine climate-adaptive management practices.

## Figures and Tables

**Table 1:** Sixteen environmental variables are involved in modeling.

Environment Variable	Abbreviation	Unit	Contribution Rate
Isothermality	bio3	1	2.713%
Max Temperature of Warmest Month	bio5	°C	24.998%
Min Temperature of Coldest Month	bio6	°C	43.542%
Temperature Annual Range	bio7	°C × 10	6.356%
Annual Precipitation	bio12	mm	10.334%
Precipitation Seasonality	bio15	1	1.355%
Elevation	alt	m	2.267%
Slope	slo	%	0.186%
Aspect	asp	°	0.037%
Available Water Capacity	awc_class	%	5.306%
Topsoil Organic Carbon	t_oc	% weight	0.315%
Topsoil pH	t_pH	−log(H+)	0.866%
Subsoil Organic Carbon	s_oc	%	0.323%
Subsoil pH	s_pH	/	0.552%
Land Use/Cover	lucc	/	0.535%
Normalized Difference Vegetation Index	ndvi	/	0.314%

## Data Availability

The datasets generated and analyzed during the current study are available from the corresponding author on reasonable request.

## Supplementary Materials

The following supporting information can be downloaded at: https://www.mdpi.com/article/10.3390/biology14111473/s1, Table S1. Accuracy evaluation of different distribution models using AUC values, TSS values, and Kappa statistics; Table S2. Current suitable habitat area of *F. tikoua* (10^4^ km^2^); Table S3. Predicted area of suitable habitats for *F. tikoua* under future climate scenarios (10^4^ km^2^); Table S4. Changes in area and proportion of the potential geographic distribution of *F. tikoua* under climate change; Table S5. Niche overlap indices and equivalence tests for *F. tikoua* under future climate scenarios.
